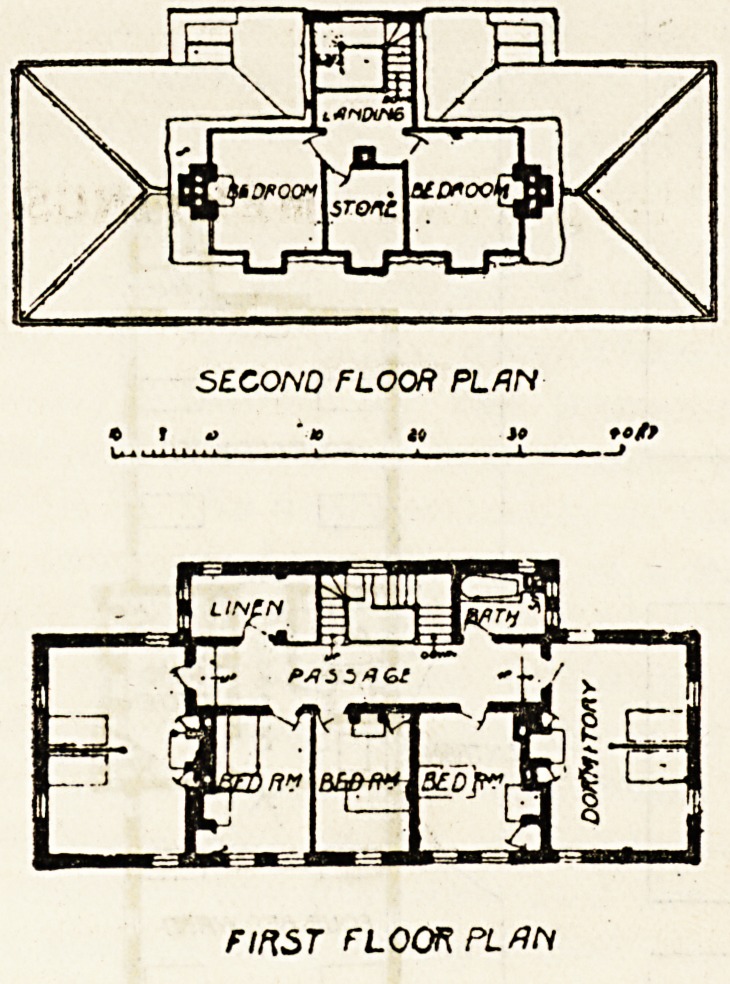# Isolation Hospital for the Ampthill District of Bedfordshire

**Published:** 1906-05-05

**Authors:** 


### ISOLATION HOSPITAL FOR THE AMPTHILL DISTRICT OF BEDFORDSHIRE.

THIS hospital was formally opened by the Duke of Bedford in December last. It provides accommodation for cases of infectious disease arising in the numerous parishes of the district. It is situate in the parish of Steppingley, about two miles from Ampthill, and one mile from Flitwick. The site has one frontage to the main road; and the hospital consists of five separate blocks, of which four form a sort of imperfect quadrangle, and the fifth—that is the laundry block—is placed at the north-east corner beyond the quadrangle.

The administrative block faces almost due south. It is a three-story building. The ground floor is divided into equal parts by a good-sized hall, on the left-hand side of which is the medical officer's room, as stated on the plan, but which is described as the matron's sitting-room in the letterpress. Then on the same side there is a lobby which gives access to the nurses' sitting-room. On the right-hand side of the hall is the scullery, having two doors, one opening into the kitchen and the other into a lobby leading to the hall. At the back of the hall is the staircase, having on one side the larder and on the other a closet.

The first floor contains two double-bedded rooms, three single-bedded rooms, a linen-room, and a bath-room, all these being for the accommodation of the matron and her staff of nurses. The second floor contains two bedrooms for domestic servants and a store-room.

The two largest pavilions lie east and west of the quadrangle. Each contains two wards for four beds each; and there is between these a ward kitchen, a passage communicating with the wards, and a bath-room lying to the right of the entrance to the pavilion. Every bed has a window on both sides, and a wall space of 12 feet. The cubic space per bed is 2,000 feet. The sanitary annexe springs from the end of the wards, and is provided with a cross-ventilated lobby. The other wards in these pavilions are exactly the same.

The four-bedded block is placed to the north. It has two wards of two beds each, and these have the same satisfactory arrangement of windows as the larger wards; and the ward kitchen occupies a similar position. This block is provided with a movable bath only. There is a verandah to the south, and the closets project from this verandah.

In addition to the ordinary washhouse and ironing-room the laundry block contains a steam disinfector by Manlove, Alliott and Co., and incorporated with this block are the mortuary, the ambulance house, and the coal-house.

The inner aspects of the ward walls are finished quite smooth, and are painted with white and green enamel. All the angles have been rounded off so as to prevent the accumulation of dust. The windows are on the double-hung sash

SECOND FLOOR PLAN

FIRST FLOOR PLAN

principle, and have "fall-in" tops for ensuring through perflation of air. The floors are laid down in teak. The wards are warmed by stoves which have chambers for heating the air before it is distributed in the rooms.

In a hospital of this size and class there is but little scope for originality in design, as the Local Government Board models and instructions are always more or less closely followed; but it may be said in this example that every part seems to have been carefully studied, and that the result is good.

The elevations are in local red brick, and the roofs are tiled. The water supply is obtained by gravitation from the Millbrook Springs, and the sewage is treated on the septic tank system.

The site was presented by the Duke of Bedford, and His Grace also contributed £500 to the cost. The rest of the money would be raised in the usual way as a charge on the rates, and the total expenditure, including furniture, etc., was not much under £10,000 for the 20 beds. The architect was Mr. Percy Adams, of Woburn Place, London; and the contractor was Mr. S. Foster, of Kempston, Bedford.

## Figures and Tables

**Figure f1:**
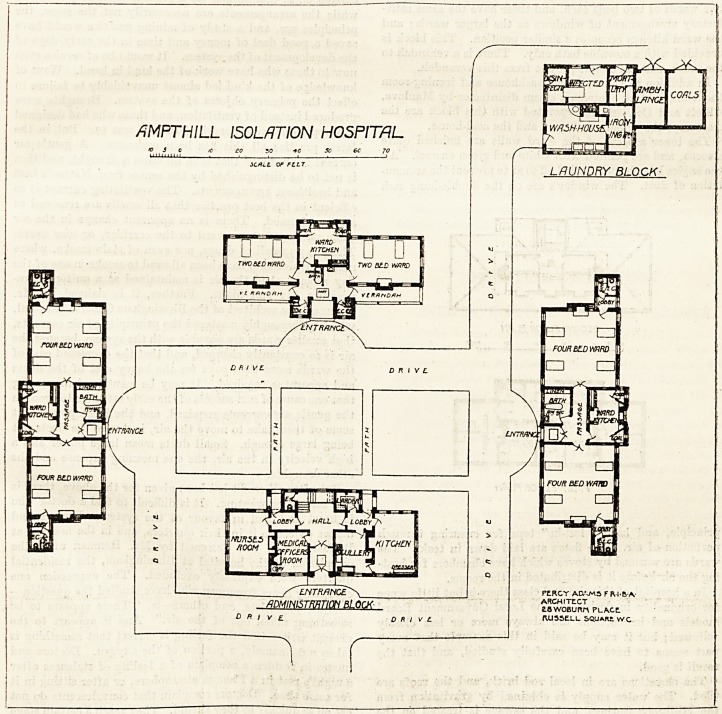


**Figure f2:**